# Proactive outreach for smokers using VHA mental health clinics: protocol for a patient-randomized clinical trial

**DOI:** 10.1186/1471-2458-14-1294

**Published:** 2014-12-17

**Authors:** Erin S Rogers, Steven S Fu, Paul Krebs, Siamak Noorbaloochi, Sean M Nugent, Radha Rao, Carolyn Schlede, Scott E Sherman

**Affiliations:** VA New York Harbor Healthcare System, 423 East 23rd Street, New York, NY 10010 USA; Department of Population Health, New York University School of Medicine, 227 East 30th Street, New York, NY 10016 USA; VA HSR&D Center for Chronic Disease Outcomes Research (CCDOR), Minneapolis VHA Health Care System, One Veterans Drive, Minneapolis, MN 55417 USA; Department of Medicine, University of Minnesota Medical School, 717 SE Delaware St, Minneapolis, MN 55414 USA; Michael E. DeBakey VHA Medical Center, 2002 Holcombe Blvd, Houston, TX 77030 USA; James A. Haley Veterans’ Hospital, 13000 Bruce B. Downs Blvd, Tampa, FL 33612 USA

**Keywords:** Smoking, Tobacco, Smoking cessation, Mental health

## Abstract

**Background:**

Persons with a mental health diagnosis have high rates of tobacco use and face numerous barriers to cessation including high levels of nicotine dependence, low rates of tobacco treatment referrals from mental health providers, and limited availability of tobacco treatment targeted to their needs. This manuscript describes the rationale and methods of a clinical trial with the following aims: 1) Compare the reach and efficacy of a proactive telephone-based tobacco cessation program for Veterans Health Administration (VHA) mental health clinic patients to VHA usual care and 2) Model longitudinal associations between baseline patient characteristics and long-term abstinence.

**Methods/design:**

We will use the electronic medical record to identify patients across four VHA healthcare facilities who have a clinical reminder code indicating current tobacco use in the past six months and who have had a mental health clinic visit in the past 12 months. We will send each patient an introductory letter and baseline survey. Survey respondents (N = 3840) will be randomized in a 1:1 fashion to intervention or control. Control participants will receive VHA usual care. Intervention participants will receive proactive motivational telephone outreach to offer tobacco treatment. Intervention participants interested in treatment will receive eight weeks of nicotine replacement therapy plus eight sessions of specialized telephone counseling over two months, followed by monthly maintenance counseling for four months. We will conduct telephone surveys with participants at six and 12 months to assess study outcomes. We will collect a mailed saliva sample from patients reporting 7-day abstinence on the telephone surveys. The primary outcome will be cotinine-validated abstinence at 12-month follow-up.

**Discussion:**

Mental health patients are a high-risk smoking population with significant barriers to cessation. This study will evaluate the efficacy of a program that proactively reaches out to smokers with a mental health treatment history to engage them into telephone cessation counseling targeted to the needs of mental health patients.

**Trial registration:**

Clinicaltrials.gov: NCT01737281 (registered November 5, 2012).

## Background

Despite considerable progress, smoking remains the leading preventable cause of death in the United States, responsible for over 480,000 deaths and $157 billion in health-related economic losses each year [1]. Persons with a mental health diagnosis (DSM-IV, Axis I or II) have particularly high rates of tobacco use and consume over 46% of cigarettes sold in the United States per year [2, 3]. Patients with bipolar disorder or schizophrenia have the highest smoking rates (69% and 58-90%, respectively) followed by those with posttraumatic stress disorder (PTSD; 45-63%) and depression (31-51%) [3–5].

Most mental health patients who smoke are interested in quitting [6–9]. Smokers with a mental health history face a number of unique barriers that increase the difficulty of quitting, namely higher nicotine dependency, shared etiology between smoking and mental health concerns, and greater susceptibility to relapse [10]. Nicotine can increase arousal, working memory, and executive functioning, which could lead to self-medication among some populations, especially those with schizophrenia, who experience particular limitations in these areas [11–13]. Higher anxiety sensitivity and negative affect avoidance may contribute to the higher relapse rates even among those who initially quit [14]. Thus, smokers with a mental health history may require specialized treatment with a focus on behavioral and affect management. In addition, current evidence suggests that increasing length of tobacco treatment follow-up may be important for preventing relapse in mental health populations [15, 16].

Even when effective tobacco treatment exists, mental health patients face barriers to accessing treatment including limited support and tobacco treatment from providers [17–20]. While physicians often assess tobacco use in mental health patients, they rarely follow-up with treatment [17]. For example, from 2006–2010 60% of outpatient psychiatry visits in the United States included tobacco screening, but psychiatrists provided tobacco cessation counseling during only 23% of visits with smokers [19]. Of particular concern is that during this same time period psychiatrists prescribed nicotine replacement therapy (NRT) during less than 1% of visits with smokers. New strategies are needed to increase access to effective tobacco cessation treatments among mental health populations.

To overcome access barriers and to better meet the needs of tobacco-dependent mental health patients, we have designed and initiated a patient-randomized controlled trial that adapts a program that was previously found to be effective at increasing access to tobacco treatment and population-level abstinence among smokers using Veterans Health Administration (VHA) primary care clinics [21]. The trial will test the efficacy of proactively reaching out to VHA mental health patients who smoke to enroll them in telephone-based counseling targeted to the needs of smokers with a mental health history. The trial has two primary aims: (1) Compare the reach and efficacy of a proactive outreach telephone-based tobacco cessation program for patients seen in mental health to usual care advice and referral to local VHA and community tobacco cessation resources and (2) Model longitudinal associations between baseline sociodemographic, medical and mental health characteristics and abstinence at six and 12 months. We hypothesize that the proactive intervention will increase the proportion of smokers who are abstinent at 12 month follow-up and that mental health distress and active substance abuse will be related to treatment uptake and long-term smoking abstinence.

## Methods/design

### Study design

**Figure 1:**
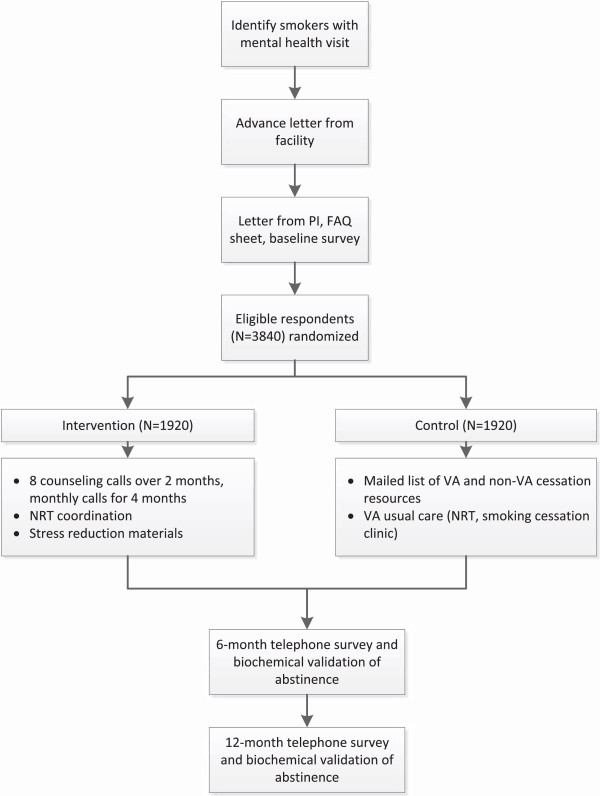
**Study overview.**

Figure 1 provides an overview of our study design. We will use the VA’s electronic medical record (EMR) system to identify a cohort of patients across four VHA health care facilities who have a clinical reminder health factor indicating tobacco use in the past six months [22] and who have had a mental health clinic visit in the past 12 months. We will send each patient an introductory letter and baseline survey. Current smokers who return a baseline survey and meet eligibility criteria (target N = 3840) will be randomized in a 1:1 fashion to intervention or control, stratified by site. Control participants will receive a mailed list of local VHA smoking cessation treatment options that they can access on their own or to which they can be referred by their regular VHA providers (i.e., usual care). Intervention participants will receive a telephone outreach call to enhance motivation and provide encouragement to start tobacco cessation treatment. Intervention participants who are interested in treatment will receive eight proactive telephone counseling sessions over two months followed by monthly relapse prevention sessions for four months. Intervention counselors will arrange for Intervention participants to receive eight weeks of NRT from one of their regular VHA providers, and they will update Intervention participants’ regular VHA mental health providers on their patients’ progress in tobacco treatment via EMR progress notes. Research assistants will conduct telephone surveys with participants at six and 12 months to assess study outcomes. Research assistants will also collect a mailed saliva sample from patients reporting 7-day smoking abstinence on the telephone surveys. The primary outcome is cotinine-validated abstinence at 12-month follow-up.

### Settings

This study will take place at four VHA facilities chosen to increase generalizability of findings by studying the intervention in four geographically and culturally diverse settings: the VA New York Harbor Healthcare System, the VA Minneapolis Healthcare System, the Michael E. DeBakey VA Medical Center in Houston, Texas and the James A. Haley Veterans’ Hospital in Tampa, Florida. The research activities taking place at participating sites were approved by the VHA Central Institutional Review Board (protocol #12-42) and each site’s Research and Development Committee.

### Identifying potential participants

Using a three-step process, we will identify potential participants using specific combinations of administrative data contained in the EMR system:

1. *Selecting current smokers:* Current smokers will be identified using EMR tobacco use clinical reminder codes, where information is stored as a health factor. Patients will be included if they have screened positive for tobacco use in the previous six months. We selected six months to reduce the false-positive rate of this sampling method. Patients will be excluded if they have an ICD 9 diagnosis of dementia (i.e., 290.XX or 331.XX).
2. *Identifying Mental Health patients:* Within the list of all eligible smokers, VHA programmers will identify patients treated in the previous 12 months in a VHA Mental Health Clinic, using VHA clinic stop codes: 502–581.
3. *Selecting the initial sample:* After receiving a list for each site of current smokers with a recent Mental Health visit, we will select all women and a random sample of men to total 1,600 potential participants from each site (N = 6,400) as our initial recruitment pool. We are selecting all women to increase the representation of women in our final sample. Since we will mail enrollment materials in batches each month during an 18-moth recruitment period, we will verify our list of eligible patients each month to ensure that their smoking status is current in the EMR at the time of enrollment. Each patient who is no longer listed as a current smoker will be replaced by another patient from the site prior to enrollment and randomization.

#### Inclusion/exclusion criteria

Using administrative data and the baseline survey, we will verify the study’s inclusion and exclusion criteria with each potential participant. Anyone who is found to be ineligible will be excluded prior to randomization and replaced with a randomly selected person from the list of current smokers for that particular site. To maximize reach and impact we have chosen broad inclusion criteria. Inclusion criteria include: (1) Current smoker (i.e., any cigarette use in past 30 days) and (2) Mental Health clinic visit in past 12 months. Exclusion criteria include: (1) ICD 9 diagnosis of dementia (excluded during data abstraction process), (2) Does not speak English, and (3) Does not have telephone and mailing address.

### Participant recruitment

We will use a modified Dillman protocol to maximize recruitment response rates and data quality [23]. First, we will send each patient a letter from the Chief of Staff, facility Behavioral Health Coordinator or other comparable leader from the facility stating the patients will soon be contacted about a smoking cessation research study with the goal of helping Veterans who use VHA mental health clinics stop smoking. The letter will also include information on how to contact the project director to opt-out of receiving further study materials or if they feel they have been contacted in error (e.g., not a current smoker or have not used mental health services in the past 12 months). One week later, we will send out a packet of information to patients, including a cover letter, a sheet of Frequently Asked Questions (FAQ) about the study that contains all elements of informed consent, and a self-administered baseline survey. The cover letter and FAQ sheet will inform potential participants that they will receive a $10 payment for returning the survey. Patients who return a mailed baseline survey and meet eligibility criteria will be enrolled in the study. The study was granted a waiver of documentation of informed consent by the VHA Central IRB.

### Randomization

When study staff receive a baseline survey, they will enter the patient’s status into a tracking system created for the study that prompts staff to verify eligibility criteria from the baseline survey. The tracking system will randomize eligible participants in a 1:1 fashion, stratified by site, using a randomization list created by the study’s statistician. Study research assistants (who conduct participant surveys, collect saliva samples, and complete other tasks such as mailing participants reimbursements) will remain blind to randomization during the study. Study counselors and supervisors will know the randomization status of each participant.

### Intervention group: proactive telephone treatment

#### Telephone counseling

Within one week of receiving a completed baseline survey, a counselor will phone participants randomized to the intervention arm. The counselor will make up to six contact attempts at different times of day to reach participants. The purpose of the outreach call is to: 1) deliver motivational enhancement to quit smoking, 2) promote self-efficacy in quitting, and 3) encourage participants to participate in smoking cessation treatment. Participants do not need to engage in counseling to participate in the study. Participants who engage in counseling will receive the full telephone counseling protocol which is adapted from a protocol we previously found to be more effective with VHA mental health patients than state Quitline counseling [24]. The protocol is characterized by:

- *Motivational enhancement* – Guided by a motivational interviewing (MI) approach, we will include motivational enhancement in each of the first several telephone calls and as-needed during later calls to increase patient motivation to quit and reduce relapse.
- *Multiple sessions* – Participants can receive up to 12 counseling calls comprised of eight calls over two months to help participants make a quit attempt followed by monthly maintenance call for four months.
- *Relapse-sensitive scheduling* – Participants will receive four calls to plan a quit date, three calls in the first two weeks after their quit date, when the relapse risk is highest, followed by another call four weeks after their quit date. Participants will receive monthly maintenance calls for four months to work through any slips or barriers to continued abstinence [15, 16].
- *Problem-solving therapy* – This approach, based on helping the smoker identify and solve expected and actual challenges, is endorsed by the national smoking cessation guidelines [25].
- *Stress Reduction* – The counseling protocol includes stress reduction techniques, such as relaxation and mindfulness exercises [26, 27], smoking schedules to remove the link between smoking and stress responses [28], and stress reduction self-help materials that counselors will discuss with participants.

#### Medication requests

All smokers in the intervention arm will be asked about their NRT preference during the outreach call and first counseling call. Their counselor will place an alert in the EMR system for their facility’s smoking cessation program or the participant’s regular primary care provider indicating that the participant expressed interest in receiving NRT and their preference. The alert will also contain relevant information from the US Public Health Services guidelines for the treatment of tobacco [25]. The counselors will monitor the participants’ EMR for whether a prescription is written. If no prescription is written within one week after sending the alert, the counselor will place a follow-up alert reiterating the participant’s expressed interest in NRT. The regular VHA providers will not be required to prescribe or be required to prescribe the patient’s preferred NRT. Rather, study counselors will simply notify the providers that the patient expressed interest in receiving NRT. Study counselors will also encourage participants to discuss NRT use with their regular providers.

#### Engaging mental health providers in the treatment process

In recognizing that a patient’s mental health providers are an important source of support and treatment encouragement for our patient population, we will engage intervention patients’ primary mental health providers into the treatment process by alerting the providers of their patients’ progress via EMR progress notes.

### Control group: VHA usual care

We will send smokers randomized to the control group a mailed list of local VHA and non-VA smoking cessation services that they can access on their own. In addition, patients randomized to the control group may receive treatment or referrals to treatment from their regular VHA providers as part of usual care. Pharmacotherapy is available at all sites in the form of nicotine replacement (patches, gum and lozenges) and bupropion.

### Measures and data collection

#### Participant surveys

Table 1 shows the study’s measures and assessment schedule. Participants will be surveyed by mail at baseline. Participants will be surveyed by telephone six and 12 months after the date study staff receive the baseline survey or by mail if they are not responsive to telephone contacts. Research assistants blinded to randomization will make up to ten attempts over a 2-month window at different days and times to reach participants for each follow-up survey. Patients will receive a $10 payment as reimbursement for completing each survey.

**Table 1:** **Measures and assessment schedule**

Measure	Baseline	6 m	12 m
**Participant surveys**			
Eligibility: Smoking in last 30 days	X		
Sociodemographics	X		
Smoking and tobacco use history	X	X	X
Nicotine dependence	X	X	X
Quit attempt history	X	X	X
Cessation treatment offered by providers	X	X	X
Texting/social media use and preferences	X		
Thoughts about quitting	X	X	X
Motivation to quit	X	X	X
Self-efficacy in quitting	X	X	X
Environmental factors	X	X	X
Physical and mental health	X	X	X
Alcohol use	X	X	X
Pain intensity and interference	X	X	X
Financial stress	X	X	X
Smoking-induced deprivation	X	X	X
**Participant saliva sample**		X	X
**Administrative data**			
Demographics			
Cessation prescriptions in prior 12 months	X		X
Healthcare utilization in prior 12 months	X		X
Body mass index in prior 12 months	X		X
Charlson comorbidity index	X		
Mental health diagnoses in prior 12 months	X		
Primary mental health diagnosis	X		
Mental health diagnostic cluster	X		

*Baseline Survey* – The baseline survey will assess sociodemographics, smoking habits and history [29], cessation treatments offered by providers in the prior 12 months, nicotine dependence [30], quitting stage of change [31], self-efficacy for smoking cessation, AUDIT-C to assess alcohol use [32, 33], Kessler-6 scale to assess psychological distress [34], PHQ-8 to assess depressive symptoms [35], texting and social media preferences for tobacco cessation, environmental factors toward smoking and quitting, pain intensity and interference, financial stress, and smoking-induced deprivation.

*Follow-up Surveys -* All measures assessed at baseline will be assessed again at six and 12 months except the sociodemographic measures and texting/social media preferences. We will assess cessation outcomes at six and 12 months using recommended guidelines for tobacco cessation clinical trials [36, 37]. We will assess smoking abstinence (7-day point prevalence), quit attempts, reduction in smoking, and use of cessation pharmacotherapy and non-pharmacological cessation treatments (including telephone counseling outside of the study).

#### Participant saliva sample

Self-reported smoking abstinence can be verified by assays of cotinine, the principal metabolite of nicotine. Participants will be eligible for verification if they report 7-day abstinence from cigarettes. Sample collection will be conducted by mail. A research assistant will send a saliva collection kit, collection instructions and a postage-paid return envelope on the day of telephone survey. Participants will receive up to three kits and five reminder calls to return a saliva sample within 30 days of completing the telephone survey. Participants will receive a $25 payment for returning a saliva sample.

#### Administrative data

We will use the EMR to obtain administrative data at baseline on all patients in the recruitment cohort and at 12 months on all participants. Data will include demographics (to use when data are missing on the baseline survey), cessation prescriptions in the prior 12 months, health care utilization in the prior 12 months, body mass index in prior 12 months, and diagnoses in the prior 12 months. We will use diagnoses in the chart to calculate a Charlson Comorbidity Index (CBI) for all participants. We will assign to each patient a primary mental health diagnosis using methods recommended by the VHA Mental Health Quality Enhancement Research Initiative (MH QUERI). Primary mental health diagnosis will be defined as the most frequently occurring diagnosis coded during mental health clinic encounters during the 12 months prior to enrolling in the trial. We will then assign one of six main diagnostic categories to each participant based on their primary diagnosis: affective disorders, substance abuse disorders, non-PTSD anxiety disorders, PTSD, schizophrenia disorders, or other diagnoses.

### Outcomes

The primary outcome will be cotinine-validated abstinence from smoking at 12-month follow-up. Secondary outcomes will be: (1) Self-reported 7-day abstinence at six and 12 month follow-up and six-month prolonged abstinence at 12 month follow-up, (2) Other cessation related outcomes at six and 12 months – e.g., quit attempts, cigarettes per day, cessation medication use, motivation, and self-efficacy, and (3) Self-reported mental health distress and active substance abuse.

### Data analysis

*Aim 1 Analysis – Intervention Reach:* We will assess the reach of proactive tobacco treatment in a VHA mental health population using two indices. First, we will calculate the proportion of patients in our recruitment cohort who respond to outreach materials and enroll in the study. This will help us understand the effectiveness of using the EMR to create a registry of smokers using VHA mental health clinics who will respond to proactive outreach. In addition, using administrative data collected on all patients in our recruitment cohort, we will use logistic regression to determine which characteristics (e.g., age, race/ethnicity, primary mental health diagnosis) are associated with response to proactive outreach. Second, among enrolled participants we will compare the two study arms on the proportion of participants who report using tobacco treatment (telephone counseling and/or medications) on follow-up surveys. We will use logistic regression to determine which characteristics from the baseline surveys are associated with use of telephone counseling and medications in the two study arms.

*Aim 1 Analysis – Intervention Efficacy:* The primary outcome for this analysis will be dichotomous cotinine-validated abstinence (cotinine <15 ng/ml) at 12 months post-enrollment [38]. The comparisons of the abstinence rates across the two groups will be made using exact logistic regression methods, accounting for stratification by site.

*Aim 2 Analysis - Modeling Longitudinal Abstinence:* We will use a generalized linear mixed model approach [39] to model abstinence at six and 12 months. Following recommended best practices in longitudinal data analysis [40], we will use a two-level hierarchical linear model to fit the data. The outcome variable of interest will be dichotomous cotinine-validated smoking abstinence. Therefore, a logit mixed model with intercept, time and time by intervention effects being random (varying across individuals), while the intervention is treated as a fixed effect. This longitudinal analytic approach will help understand to what extent treatment effects differ between six and 12 months and whether they show a delayed effect (either increasing or decreasing over time) to determine a treatment main effect as well as a treatment by time interaction. Since this is a randomized experiment across the treatment arms, no covariate adjustments is needed. However, in the cases that randomization is not successful to provide balance, imbalanced baseline covariates will be included at the patient level (e.g., age, sex, race/ethnicity, nicotine dependence, motivation, self-efficacy, mental distress, and current substance use).

#### Missing data

We anticipate low, but potentially important rates of survey non-response to all or part of the 6- and 12-month surveys. We will use the evidence-based recruitment strategies described above (reimbursements, reminders, mixed mode survey administration) to minimize non-response. A common practice in smoking cessation research is to treat non-respondents at follow-up as continuing smokers (i.e., intent to treat). This practice is perceived to be a conservative approach but does not produce valid estimates of quit rates [41]. Therefore, we will use multiple imputations to fully use available baseline information and partial later surveys. We will use a propensity-based multiple imputation method similar to that discussed by Little [42] using separate imputation procedures within each study group and design stratum combination. Within a given combination, we will estimate the propensity for responding to cotinine validated abstinence for each individual from a logistic regression model for survey response using the characteristics measured at baseline as explanatory measures. Within a combination of intervention and design strata, we will further stratify the sample according to the values for these estimated propensities. Within a propensity substratum, we will impute a value for the outcome measure for each non-respondent by randomly sampling an outcome value from the respondent values in the substratum. Multiple completed datasets will be created and the point estimates and the estimated standard error from each dataset will be combined to arrive at a single point estimate (using the specific method discussed below), its estimated standard error, and the associated confidence interval or significance test. This approach assumes that missing outcome data due to non-response is missing at random.

To assess the impact of non-ignorable survey non-response, or missing not at random non-response, we will implement pattern-mixture analyses. Using content expertise and the observed missing data patterns, we will develop these pattern-mixture models. The nature of these models is difficult to specify in advance of observing the different patterns but we will posit distributions for the missing data. For each set of posited distributions and the observed data, we will calculate revised estimates for the relevant intervention effects. One potential model would be to use the propensity stratum derived in the analysis described above and, within each stratum build a distribution for the outcome of interest by shifting the observed distribution among the responders. Content expertise and empirical results will be used to determine the form and magnitude of shift. These distributions would then be used to impute values for the non-responders to cotinine validation in the imputation process described immediately above. Variations of this approach and other approaches will be used to assess the sensitivity of the analyses above to non-ignorable non-response.

#### Power calculation

The power analysis is based on the primary outcome – cotinine-validated abstinence at 12-month follow-up. Our data are stratified on four hospital sites. Considering that a stratified random sample is usually more efficient than a simple random sample, we assume the two groups are two independent simple random samples. We estimate the quit rate in the control group will be 4%. In the intervention group, we estimate that about 20% of participants will use telephone counseling and that the quit rate among Veterans enrolled in counseling will be 16%. When the treatment cessation rate is combined with that from patients who do not accept treatment (assuming also a 4% quit rate), there will be a population-level quit rate of 6.4% in the intervention group. A sample size of 3840 (N = 1920 per arm) provides 80% power to detect any increase greater than 2.0% in population-level abstinence rate for the intervention group when the abstinence rate in the control group is 4%, a small but clinically-meaningful quit rate that results in overall cost-savings [43].

## Discussion

Persons with a mental health diagnosis use tobacco at alarming rates, which has a large negative impact on their health and quality of life. Their increased vulnerabilities to tobacco dependence, high risk for relapse, and difficulties in accessing tobacco treatment make them an important population to target with tobacco cessation interventions. The present study examines the feasibility of an innovative health care delivery model designed to overcome barriers to care and maximize the utility of the electronic health records to target effective tobacco cessation treatment to the entire population of mental health smokers in four VHA healthcare systems.

## Abbreviations

AUDIT-C: Alcohol use disorders identification test
CBI: Charlson comorbidity index
DSM-IV: Diagnostic and statistical manual, 4th edition
EMR: Electronic medical record
FAQ: Frequently asked questions
ICD-9: International classification of diseases, 9th edition
MH QUERI: VHA mental health quality enhancement research initiative
MI: Motivational interviewing
NRT: Nicotine replacement therapy
PHQ-8: Personal health questionnaire, 8-item version
PTSD: Post-traumatic stress disorder
VHA: Veterans health administration.
